# Preventing infection transfer from health facilities to rural communities

**DOI:** 10.1186/2047-2994-4-S1-P253

**Published:** 2015-06-16

**Authors:** CO Nwuba, NF Onyemelukwe, E Agbata

**Affiliations:** 1 Medical Laboratory Sciences, University of Nigeria, Enugu, ENUGU, Nigeria

## Introduction

In developing countries especially sub Saharan Africa, the pressure of increased population has led to increasing new concerns for a proper and affordable waste disposal treatment in health care facilities. Effluents of hospital wastes are discharged into streams which are the major source of water supply for inhabitants of most communities. The non-existence of portable water supply in most communities has led to dependence on these streams for drinking, irrigation and other domestic chores which in turn results in outbreak of gastrointestinal infections in surrounding communities.

## Objectives

To examine the impact of infectious waste transfer from health facilities to rural communities

## Methods

Fecal samples and sewage obtained from effluent streams adjacent to health facilities, ill maintained waste treatment plants and control samples from patients at nearby health facilities were screened for parasitic ova, cyst and larva. Graded doses of Calcium Hydroxide (Ca (OH)_2_) was added into these samples and re-screened for parasites.

## Results

Distribution of parasites in effluent streams and waste plants adjacent to health facilities showed significant occurrence (P<0.05) of C. sinensis (38.4%), A. lumbricoides (23.1%), T. trichura (33.3%), S. stercoralis (44.4%) and I. belli (33.3%) respectively. Lime (Ca (OH)_2_) treatment caused a significant reduction in the number of parasites at PH 8.0 and 10.0. Higher PH, increase in temperature and longer exposure to pretreatment with (Ca (OH)_2_) were observed as important factors for parasite eradication in these samples.

## Conclusion

Inefficient waste disposal systems, non-functional waste treatment plants, poor supply of portable water as well as government’s general neglect of health issues has greatly increased enteric diseases in developing countries. Hence it is necessary that human waste be properly treated to eliminate all pathogenic organisms before disposal. Lime Stabilization technique has proven to be a simple, safe, cost effective, easily available and environmentally protective alternative model for waste treatment especially in resource constrained settings. It is worth suggesting in the light of this work that lime be added in sufficient doses into septic tank of hospitals before disposal in order to avoid contamination by parasites including residuals reaching effluent streams.

## Disclosure of interest

None declared.

